# Gaussian Fuzzy Number for STR-DNA Similarity Calculation Involving Familial and Tribal Relationships

**DOI:** 10.1155/2018/8602513

**Published:** 2018-07-29

**Authors:** Maria Susan Anggreainy, M. Rahmat Widyanto, Belawati H. Widjaja, Nurtami Soedarsono

**Affiliations:** ^1^Faculty of Computer Science, Universitas Indonesia, Depok Campus, West Java 16424, Indonesia; ^2^Faculty of Dentistry, Universitas Indonesia, Salemba Campus, Jakarta 10430, Indonesia

## Abstract

We performed locus similarity calculation by measuring fuzzy intersection between individual locus and reference locus and then performed CODIS STR-DNA similarity calculation. The fuzzy intersection calculation enables a more robust CODIS STR-DNA similarity calculation due to imprecision caused by noise produced by PCR machine. We also proposed shifted convoluted Gaussian fuzzy number (SCGFN) and Gaussian fuzzy number (GFN) to represent each locus value as improvement of triangular fuzzy number (TFN) as used in previous research. Compared to triangular fuzzy number (TFN), GFN is more realistic to represent uncertainty of locus information because the distribution is assumed to be Gaussian. Then, the original Gaussian fuzzy number (GFN) is convoluted with distribution of certain ethnic locus information to produce the new SCGFN which more represents ethnic information compared to original GFN. Experiments were done for the following cases: people with family relationships, people of the same tribe, and certain tribal populations. The statistical test with analysis of variance (ANOVA) shows the difference in similarity between SCGFN, GFN, and TFN with a significant level of 95%. The Tukey method in ANOVA shows that SCGFN yields a higher similarity which means being better than the GFN and TFN methods. The proposed method enables CODIS STR-DNA similarity calculation which is more robust to noise and performed better CODIS similarity calculation involving familial and tribal relationships.

## 1. Introduction

Genetics is the study of genes, genetic variation, and heredity in living organisms. Population genetics is a part of evolutionary biology and is a subfield genetic that deals with genetic differences within and between populations [1]. Variations in traits among human populations represent genetic differences that can be inherited from generation to generation. Population genetics is learning about genetic variation in the population, involving the examination and modeling of changes in the frequency of genes and alleles in populations over time and space [2]. Population genetics gives us the opportunity to step back and observe patterns of genetic change over time. Comparing populations to one another can lead to capturing how external factors trigger the evolution of a trait, as well as mapping variants associated with various traits within the population. Population genetics is another way of looking at DNA that can generate insight into the potential to benefit everyone. Many of the genes found in a population will be polymorphic, that is, will occur in a number of different alleles. Mathematical models are used to investigate and predict the occurrence of specific alleles or combinations of alleles in the population; the focus is by comparing data groups or populations or species, not individuals.

A population is a group of individuals with the same characteristics (species) that live in the same place and have the ability to reproduce among each other; evolution also works through populations [3]. Geneticists, on the other hand, view the population as a means or container for the exchange of alleles owned by the individuals of its members. The dynamic frequency of alleles in a population is of major concern in the study of population genetics

DNA regions with short repeat units (usually 2-6 base pairs in length) are called Short Tandem Repeats (STR). STRs are found surrounding the chromosomal centromere. STRs have proven to have several benefits that make them especially suitable for human identification [4]. STRs have become popular DNA markers because they are easily amplified by polymerase chain reaction (PCR) without the problem of differential amplification; that is, the PCR products for STRs are generally similar in amount, making analysis easier. An individual inherits one copy of an STR from each parent, which may or may not have similar repeat sizes. The number of repeats in STR markers can be highly variable among individuals, which make these STRs effective for human identification purposes [5].

Beginning in 1996, the FBI Laboratory launched a nationwide forensic science effort to establish core STR loci for inclusion within the national database known as CODIS (Combined DNA Index System). The 13 CODIS loci are CSF1PO, FGA, TH01, TPOX, VWA, D3S1358, D5S818, D7S820, D8S1179, D13S317, D16S539, D18S51, and D21S11. These loci are nationally and internationally recognized as the standard for human identification. DNA STR markers used in this research were 15 CODIS loci with two additional loci, i.e., D19S433, and D2S1338 has additional loci for an extensive and powerful STR testing battery if required [6, 7].

A person's DNA profile can match DNA profile data similarity to another person. DNA profile plays an important role in solving problems related to the family's father and other family members [8, 9]. This method is a way that is legally used for solving to prove the validity of kinship or family ties of the person, identifying unknown body of war or natural disaster victims, and studying human population [10, 11].

In previous research, it has been noted that although M. R. Widyanto et al. [12–14] are quite sufficient in setting with triangular fuzzy number similarity of the size between the two alleles, the statistical information on the ethnicity of the two profiles' information is lost. To overcome the problem, this research employs novel methods to measure similarity between tribes that gives a better result than previous method. We proposed shifted convoluted Gaussian fuzzy number (SCGFN) and Gaussian fuzzy number (GFN) to represent each locus value as improvement of triangular fuzzy number (TFN) as used in previous research.

Research method was proposed in Section 2. Experimental results on three methods are shown in Section 3. Analyses of statistical and comparison tests are summarized in Section 4.

## 2. Proposed Research Method

### 2.1. Fuzzy Sets

Fuzzy sets are held as a basis for the theory of possibility. A fuzzy set A in x is formally defined as follows [15]:

(1) $$\begin{array}{c} A=\left\{\;\left(\;x,\mu_{A}\left(\;x\;\right)\;\right)\;|\;x\;\varepsilon X\;\right\} \end{array}$$

where x is the universe of discourse and is the membership degree of the x in A. When fuzzy set theory was presented, researches considered decision-making as one of the most attractive application fields of that theory [16].

### 2.2. Measurement of Similarity Values of Two-Individual STR-DNA

**Figure 1:**
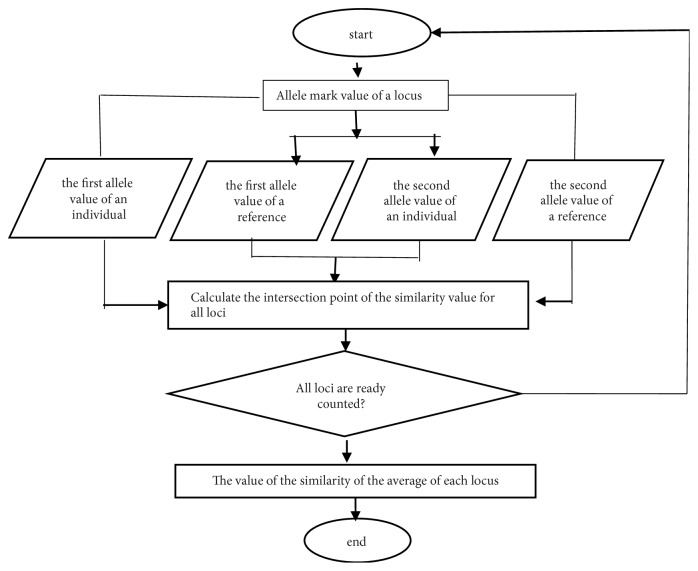
Calculation flow of similar two individuals.

The calculation to find the STR-DNA similarity of two individuals (as shown by Figure 1) is the STR-DNA value of allele 1 of the individual in comparison with the allele value 1 STR-DNA of the reference and the STR-DNA value of allele 2 of the individual with the STR-DNA value of allele 2 of each reference locus. Then we find the intersection point value of the two alleles for each locus and then calculate the average value of the similarity of each locus.

### 2.3. Two-Individual Matching: Evidence versus Reference with TFN Similarity

A triangular fuzzy number (TFN) *α* can be defined by a triplet (*a*1, *a*2, and *a*3). The triangular fuzzy number is used to represent uncertainty resulting from imprecision of polymerase chain reaction (PCR) machine. The membership function *μa*(*x*) is [17]

(2) $$\begin{array}{c} \mu_{a}\left(\;x\;\right)=\left\{\begin{array}{cc} 0, & x<a_{1},a_{3}<x\\ \frac{x-a_{1}}{a_{2}-a_{1}}, & a_{1}\leq x\leq a_{2}\\ \frac{x-a_{3}}{a_{2}-a_{3}}, & a_{2}\leq x\leq a_{3} \end{array}\right. \end{array}$$

where 0 ≤ *a*1 ≤ *a*2 ≤ *a*3 ≤ 1,  *a*1 and *a*3 stand for the lower and upper values of the support of *α*, respectively, and *a*2 stands for the modal values. Value of every DNA loci is represented by fuzzy triangular number where the fuzziness value is set to be 0.4 through experiments and the center of the fuzziness is the value of the corresponding loci. The similarity value between an allele of DNA profile evidence and DNA profile reference is given by

(3) $$\begin{array}{c} t=\frac{a3-a1}{2\left(a3-a2\right)} \end{array}$$

where the value of the first allele < value of the second allele; t is intersection of the two alleles,

a2 is STR-DNA value of the first allele, a3 is a2 + 0.2, and a1 is STR-DNA value of the second allele -0.2.

If t is a result of a negative value calculation, then t is considered zero because it means there is no intersection on both STR values; therefore, t values stay at interval [0,1].

**Figure 2:**
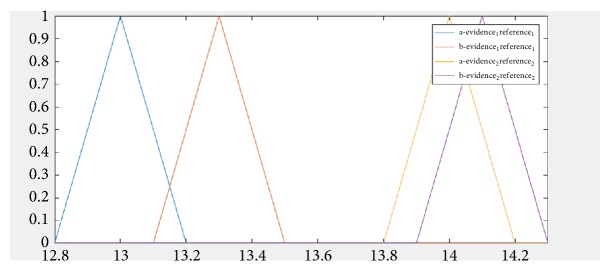
The fuzzy triangular number.

The following example shows the geometric calculation of the individual intersection points and the reference (as shown by Figure 2) with the D8s1179 locus where the allele values are individual STR-DNA = 13 and the allele value of one in reference STR-DNA = 13.3 and the allele value of two on the individual STR-DNA = 14 and the value of the two alleles in the reference STR-DNA = 14.1.

The similarity between two DNA alleles is thus calculated as the average of the similarity of the entire locus, which in turn is arithmetic mean, which is expressed as

(4) $$\begin{array}{c} t_{i}=\frac{\sum_{j=1}^{N}\mu \left(x_{i},y_{j}\right)}{N} \end{array}$$

where *t*_*i*_ is the value of similarity between DNA profile individual and DNA profile reference of the *i*th individual, *x*_*i*_ is a vector of DNA profile individual, and *y*_*j*_ is a vector of DNA profile reference. The vectors *x*_*i*_ and *y*_*j*_ are the *N* (∈*N*)-dimensional vector consisting of the value of 15 loci without amelogenin as has been used by Federal Bureau of Investigation (FBI).

### 2.4. Two-Individual Matching: Evidence versus Reference with GFN Similarity

To improve the capability of locus matching, we propose GFN (Gaussian fuzzy number) similarity. Compared to traditional triangular fuzzy number (TFN), GFN is more realistic to represent uncertainty of locus information because the distribution is assumed to be Gaussian. The Gaussian fuzzy function transforms the original values into a normal distribution. The midpoint of the normal distribution defines the ideal definition for the set, assigned a 1, with the remaining input values decreasing in membership as they move away from the midpoint in both the positive and negative directions. The input values decrease in membership from the midpoint until they reach a point where the values move too far from the ideal definition and definitely not in the set and are therefore assigned zeros. The fuzzy Gaussian function is given below [18]:

(5) $$\begin{array}{c} f\left(\;x\;\right)=ae^{-{\left(x-\mu_{f}\right)}^{2}/2\sigma_{f}^{2}} \end{array}$$

A Gaussian Membership function is specified by two parameters: a Gaussian membership function is determined complete by *μ* and *σ*; *μ* represents the membership ship center (the peak of the curve) and *σ* determines the membership function width.

Intersection from two Gaussian functions is as follows [19]:

(6) $$\begin{array}{c} v=\left\{\begin{array}{cc} 1 & if\;\;\mu 2\leq \mu 1\\ \exp\left[-{\left[\left(\frac{\mu 1-\mu 2}{\sigma 1+\sigma 2}\right)\right]}^{2}\right] & if\;\;\mu 2<\mu 1 \end{array}\right. \end{array}$$

where *μ*1 is value of STR-DNA from individual, *μ*2 is value of STR-DNA from reference, *σ*1 is the sigma value of the individual, and *σ*2 is the sigma value of the reference.

**Figure 3:**
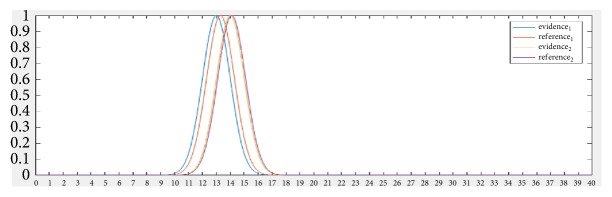
Fuzzy Gaussian similarity.

The following example shows Gaussian of the individual intersection points and the reference (as shown by Figure 3) with the D8s1179 locus where the allele values are individual STR-DNA = 13 and the allele value of one in reference STR-DNA = 13.3 and the allele value of two on the individual STR-DNA = 14 and the value of the two alleles in the reference STR-DNA = 14.1.

### 2.5. Two-Individual Matching: Evidence versus Reference with SCGFN Similarity

To improve the capability of locus matching in which ethnic information is involved, we propose SCGFN (shifted convoluted Gaussian fuzzy number) similarity. The original Gaussian fuzzy number (GFN) is convoluted with distribution of certain ethnic locus information. Therefore, the new SCGFN more represents ethnic information compared to original GFN.

The convolution function is a multiplication of the individual fuzzy Gaussian locus function and the fuzzy Gaussian approximation of the population locus. The fuzzy Gaussian function of the population locus approximation is obtained by extracting the mean value at which the mean value of the fuzzy Gaussian population locus is the STR-DNA value of the most population density and deviation of the particular population. Fuzzy Gaussian individual locus obtained, where the mean is the STR-DNA value of an individual locus, with the standard deviation value is the mean value minus 2.

The convolution is a mathematical operation on two functions (f and g) to produce a third function, that is, typically viewed as a modified version of one of the original functions, giving the integral of the pointwise multiplication of the two functions as a function of the amount that one of the original functions is translated [20]. The function f is obtained from the individual and the function g is derived from the reference

(7) $$\begin{array}{c} f\left(\;x\;\right)=ae^{-{\left(x-\mu_{f}\right)}^{2}/2\sigma_{f}^{2}}; \end{array}$$

and

(8) $$\begin{array}{c} g\left(\;x\;\right)=ae^{-{\left(x-\mu_{g}\right)}^{2}/2\sigma_{g}^{2}} \end{array}$$

convolution operator is [21]

(9) Pf⊗gx=F−1FfxFgx=ae−x−μf+μg2/2σf2+σg2

where a is the height of fuzzy = 1. For counting means,

(10) $$\begin{array}{c} \mu_{f⊗g}\left(\;x\;\right)=\mu_{f}+\mu_{g} \end{array}$$

and standard deviation is as follows:

(11) $$\begin{array}{c} \sigma_{f⊗g}\left(\;x\;\right)=\sqrt{\sigma_{f}^{2}+\sigma_{g}^{2}} \end{array}$$

The convoluted fuzzy number will replace the fuzzy number as individual locus representation value. Therefore, to get a stronger tribal relationship individual similarity calculation value is involved then the convoluted Gaussian fuzzy number is shifted approaching to the tribal population reference fuzzy number. The new shifted convoluted fuzzy number is called shifted convoluted Gaussian fuzzy number (SCGFN). This SCGFN is a new representation value of individual locus. Obtaining the mean value of SCGFN is to compare the mean value of individual fuzzy Gaussian (*μ*_*i*_) with the mean value of fuzzy Gaussian approximation of the locus population (*μ*_*ip*_). And then, the convoluted fuzzy number is shifted approaching to the fuzzy Gaussian approximation of locus population of certain tribe. The algorithm for shifting SCGFN is given below:

(12) if  μip<μiend;μscgfn=μi−0.02 ∗ μip−μi;end;endSS;elseif  μip>μiend;μscgfn=μi+0.02 ∗ μip−μi;elseend;μscgfn=μi;end;

The standard deviation value of SCGFN is the sum of the standard deviation value of the individual fuzzy Gaussian number (*σi*) with the standard deviation value of the fuzzy Gaussian approximation of the population locus of certain tribe (*σip*). The following formula is for the SCGFN standard deviation:

(13) $$\begin{array}{c} \sigma_{scgfn}=\sigma_{ip}+\sigma_{i} \end{array}$$

### 2.6. Measuring Tribal Relative Value from a DNA Profile

**Figure 4:**
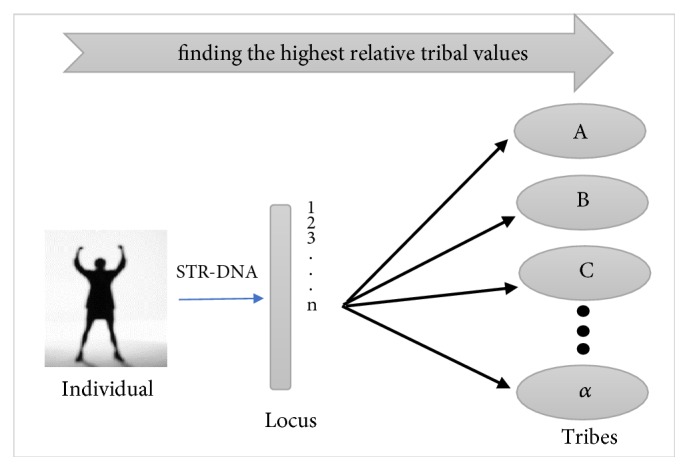
Tribal inference system architecture design.

In general, the work flow of the tribal inference system is to find the value of the tribal similarity done by calculating the average value of the point of intersection of the value of the individual similarity to the value of the tribal population in the database of 15 loci. The tribe having the highest similarity value to the individual profile will be selected as the ethnic estimation of the profile. Workflow process can be seen in Figure 4.

Tribal matching with triangular fuzzy number is obtained from the intersection of fuzzy triangular individual and triangular fuzzy population approximation. From each tribal population the mean intersection value of the individual fuzzy triangular and fuzzy triangular population approximation for each locus are calculated, and to determine the ethnic population of the individual the greatest value of the mean value of each locus of a tribal population triangular fuzzy individuals is obtained by using formula (3). Fuzzy triangular population approximation is also obtained by using formula (3) where a2 is the STR-DNA value of the largest population of density, a3 is a2 + 0.2, and a1 is a2 - 0.2.

Example is shown in Table 1.

The output for population A is at locus D3S1358 and on allele 1. The value of 13th, 14th, 15th, 16th, 17th, and 18th STR-DNA are 3, 10, 27, 31, 8, and 1 individuals, respectively, as the 16th STR-DNA shows the most number of individuals at 31, it can be concluded that the value of a1 = 15.8, a2 = 16, and a3 = 16.2.

### 2.7. Tribal Matching with GFN

Tribal matching with Gaussian Fuzzy Similarity was obtained from individual fuzzy Gaussians with fuzzy Gaussian population approximation. Gaussian fuzzy individuals are obtained by using equation of formula (7), where *μ*_*f*_ is individual STR-DNA value and *σ*_*f*_ = 1. Gaussian fuzzy population is obtained by using equation of formula (7), where *μ*_*f*_ means the distribution of the number of individuals and *σ*_*f*_ is the standard deviation from the distribution of the number of individuals. Calculation of standard deviation is as follows:

(14) $$\begin{array}{c} s=\frac{\sqrt{n\sum x_{i}^{2}-\sum {\left(x_{i}\right)}^{2}}}{n\left(n-1\right)} \end{array}$$

where *n* is number of STR-DNA values and *x*_*i*_ is the number of individuals of a locus population.

### 2.8. Tribal Matching with SCGFN

Shifted convoluted Gaussian fuzzy number (SCGFN) will replace the individual fuzzy number. SCGFN is a new individual locus with increasingly strong ethnicity. Tribal matching with SCGFN is calculating the value of similarity intersection between the corresponding SCGFN and population's fuzzy number as in tribal matching in GFN. Then look for the maximum value for all tribes. The maximum tribal value means the individual's tribal value.

## 3. Experimental Results

The DNA profile data to be entered into the database system is a PCR based DNA identification profile consisting of 15 loci, excluding amelogenin, each consisting of two alleles for each locus. The DNA profile used as an input to a tribal inference system is an Indonesian DNA with a total of 240 DNA data comprising Java (A), Malay (B), Mentawai (C), and Toraja (D) tribes. The experiments have been done using the Matlab R2016b. The experiment was conducted with four cases.

### 3.1. Same Person

**Figure 5:**
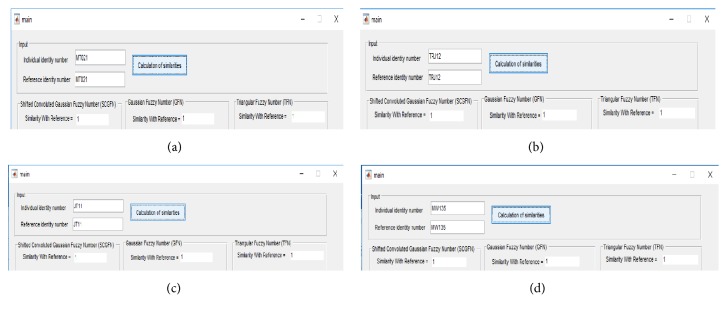
Examples from the same person similarity calculation.

From 240 pieces of data experiments were conducted with the same people, with SCGFN, GFN, and TFN; the similarity values are equal 1.  Figure 5 is an example of whether the individual identity and reference entered are the same person and the result of the similarity obtained is 1:

- Input individual id MT021 and reference id number MT021
- Input individual id TRJ12 and reference id number TRJ12
- Input individual id JT11 and reference id number JT11
- Input individual id MW135 and reference id number

### 3.2. People Who Have Family Relationships

DNA profiles tested in both biological parents are father and mother. From the experiment, the average individual similarity with family reference is obtained: SCGFN 87%, TFN 39%, and GFN 74%.  Table 2 shows ten instances of the result of the similarity of an individual with a reference being a mother or father.

### 3.3. People Who Belong to the Same Tribe

From the experiment, the average similarity of two individuals who have the same tribe is obtained: SCGFN 89.6%, TFN 21%, and GFN 65.14%.  Table 3 shows ten instances of the result of the similarity of two individuals from the same tribe.

### 3.4. Certain Tribal Populations

**Figure 6:**
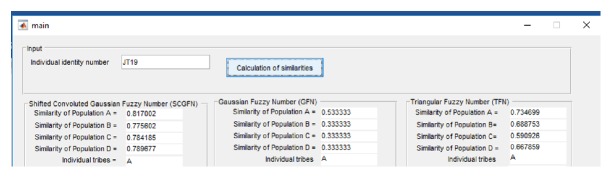
Examples of tribal population certain.

Population data consists of four tribes where the number of people in tribe A is 80, the number of people in tribe B is 100, the number of people in tribe C is 20, and the number of people in tribe D is 40.  Figure 6 shows an example of the tribal population similarity calculation of the individual identity JT19. From Figure 6 it can be seen that SCGFN, GFN, and TFN displaying the tribe of JT11 are tribe A.

Table 4 shows the result of similarity values with a certain tribal population with SCGFN, TFN, and GFN. From 80 experiments on the A tribe, the average tribe population was found to be 79% with fuzzy convolution, 45% with fuzzy triangular, and 71% with fuzzy Gaussian. The average B population of 100 experiments were 81% with fuzzy convolution, 46% with fuzzy triangular, and 76% with fuzzy Gaussian. The average C population of 40 experiments were 80% with fuzzy convolution, 45% with fuzzy triangular, and 73% with fuzzy Gaussian. The average D population of 20 experiments were 84% with fuzzy convolution, 64% with fuzzy triangular, and 79% with fuzzy Gaussian.

## 4. Analysis of Statistical and Comparison Tests

To perform analysis of the test results that have been done, statistical tests were used. To know the difference of average value of STR-DNA similarity value of the three methods used in this study, ANOVA and comparative test were used. Interpretation of ANOVA test is that if the test results show that H0 failed to be rejected (no difference), then post hoc test is not done. Conversely, if the test results indicate H0 is rejected (there is a difference), then a post hoc advanced test should be performed. To give a clear explanation why SCGFN is better than GFN and TFN, analysis of statistical and comparison test with Tukey method in ANOVA is provided. Statistical tests were performed using Minitab 16. Statistical tests were performed for 3 cases.

### 4.1. Individuals Who Have Family Relationships

To find out the different methods used in this method, we used ANOVA and comparative tests. In the one-way ANOVA test, there is only one independent variable for this case as independent variables are individuals who have family relationships. The summary of variance analysis in Algorithm 1 was obtained; P value ≤ 0.001. Associated with the level of significance (*α*) = 0.05, obtained p <*α* means H0 is rejected so it can be concluded that there is a difference between the three methods. To determine which method is better than the other method, it is further tested by the Tukey method.

In Algorithm 2 it can be seen that

- TFN < SCGFN, because it does not contain zero and center negative;
- GFN < SCGFN, because it does not contain zero and center negative;
- GFN > TFN, because it does not load zero and center positive.

**Figure 7:**
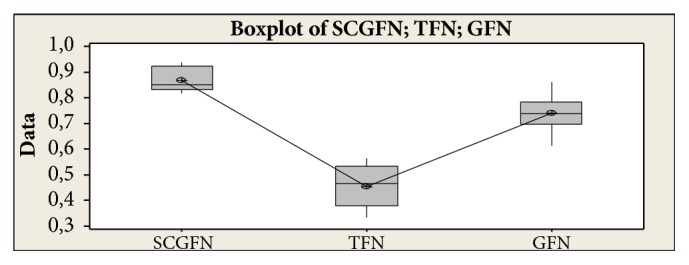
Boxplot people who have family relationships.

Then it can be concluded that TFN < GFN < SCGFN. From the result of similarity test with three methods and boxplot obtained in Figure 7, it can be seen that SCGFN method used in this research has higher similarity value in comparison with GFN and TFN method.

### 4.2. Individuals Who Belong to the Same Tribe

The summary of variance analysis in Algorithm 3 was obtained; P value ≤ 0.001. Associated with the level of significance (*α*) = 0.05 or confidence level 95%, obtained p <*α* means H0 is rejected so it can be concluded that there is a difference between the three methods. To determine which method is better than the other method, it is further tested by the Tukey method.

In Algorithm 4 it can be seen that

- TFN < SCGFN, because it does not contain zero and center negative;
- GFN < SCGFN, because it does not contain zero and center negative;
- GFN > TFN, because it does not load zero and center positive.

**Figure 8:**
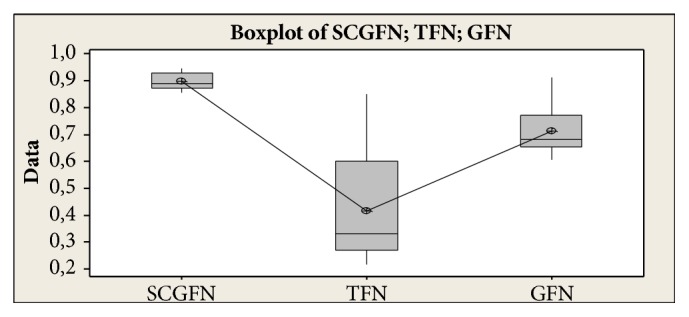
Boxplot Individuals who belong to the same tribe. Comparison test with Tukey method.

Then it can be concluded that TFN < GFN < SCGFN. From the result of similarity test with three methods and boxplot obtained in Figure 8, it can be seen that SCGFN method used in this research has higher similarity value in comparison with GFN and TFN method.

### 4.3. Certain Tribal Populations

Testing is done with 720 data, that is, 240 data with 3 methods. The summary of variance analysis for A, B, C, and D in Algorithm 5 was obtained; P value ≤ 0.001. Associated with the level of significance (*α*) = 0.05, obtained p <*α* means H0 is rejected so it can be concluded that there is a difference between the three methods.

The experiment was conducted with 3 methods, where the method of SCGFN is method 1, TFN method is method 2, and GFN is method 3. To determine which method is better than other method then each tribe is tested further. In Algorithm 6 the comparison of three methods in ANOVA test results in tribal population A can be explained:

1. TFN < SCGFN because it does not load zero and center negative.
2. GFN < SCGFN because it does not load zero and center negative.
3. GFN > TFN because it does not contain zero and positive center.

So it can be concluded that TFN < GFN < SCGFN which means SCGFN is better than GFN and GFN is better than TFN.

In Algorithm 7 the comparison of three methods in the tribal population B can be explained:

1. TFN < SCGFN because it does not load zero and center negative.
2. GFN = SCGFN because it loads zero.
3. GFN > TFN because it does not contain zero and positive center.

So it can be concluded that TFN < (GFN = SCGFN), which means that SCGFN and GFN methods are equal and better than TFN.

In Algorithm 8 the comparison of 3 methods in tribal population C can be explained:

1. TFN < SCGFN because it does not load zero and center negative.
2. GFN < SCGFN because it does not load zero and center negative.
3. GFN > TFN because it does not contain zero and positive center.

So it can be concluded that TFN < GFN < SCGFN which means SCGFN is better than GFN and GFN is better than TFN.

In Algorithm 9 the comparison of three methods in the tribal population D can be explained:

1. TFN < SCGFN because it does not load zero and center negative.
2. GFN = SCGFN because it loads zero.
3. GFN > TFN because it does not contain zero and positive center.

So it can be concluded that TFN < (GFN = SCGFN), which means that SCGFN and GFN methods are equal and better than TFN.

## 5. Conclusions

In this research, the experiments were conducted to find a better method to obtain higher individual similarity values and to find stronger tribal properties. To improve the capability of locus matching, SCGFN and GFN have been proposed. It performed fuzzy number similarity of the size between the two alleles. Experiments were done for the following cases: people with family relationships, people of the same tribe, and certain tribal populations. In these three cases, ANOVA shows the difference in similarity between SCGFN, GFN, and TFN with a significant level of 95%. In the case of people with family relationship and the case of people of the same tribe with Tukey method in ANOVA shows that SCGFN yields a higher similarity which means better than the GFN and TFN methods. While in the case of certain tribal population with Tukey method in ANOVA shows in population A and population C, SCGFN better than GFN and TFN, whereas, in population B and population D, SCGFN is equal to GFN and better than TFN. The proposed method enables CODIS STR-DNA similarity calculation which is more robust to noise and performed better CODIS similarity calculation involving familial and tribal relationships.

## Figures and Tables

**Algorithm 1:**
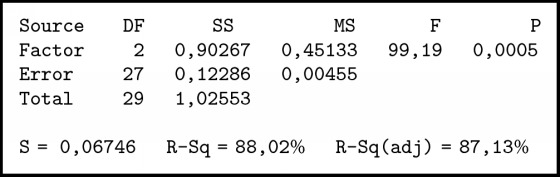
ANOVA individual test results of those who have family relationship.

**Algorithm 2:**
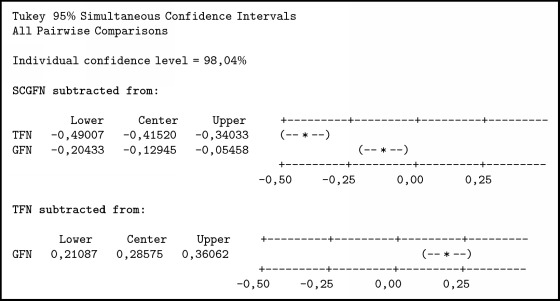
Comparison test with Tukey method.

**Algorithm 3:**
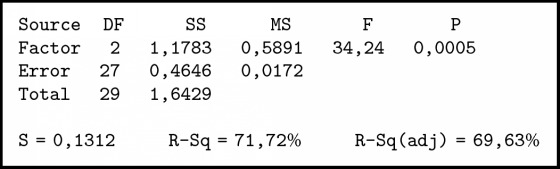
Individual test results with the same tribe using ANOVA.

**Algorithm 4:**
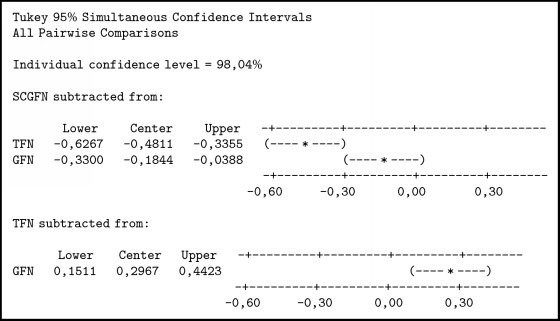
Comparison test with Tukey method.

**Algorithm 5:**
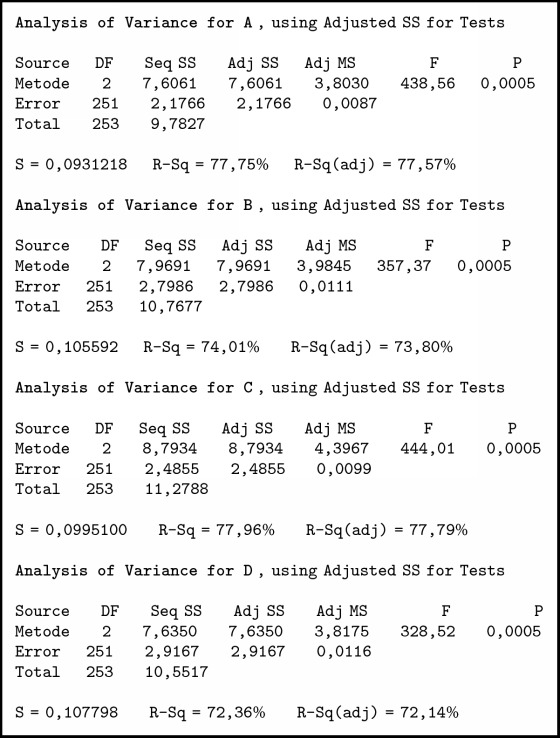
ANOVA test results for a particular tribe.

**Algorithm 6:**
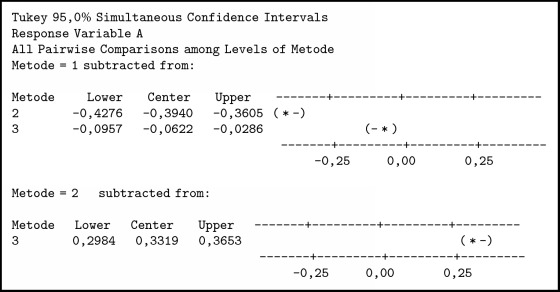
ANOVA test results on tribal population A.

**Algorithm 7:**
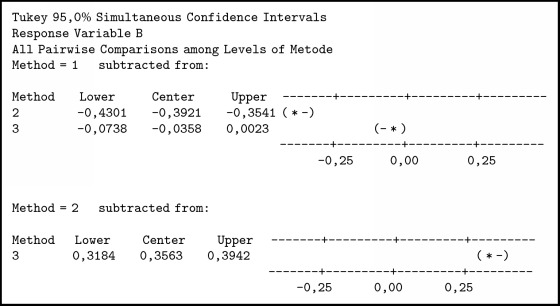
ANOVA test results on tribal population B.

**Algorithm 8:**
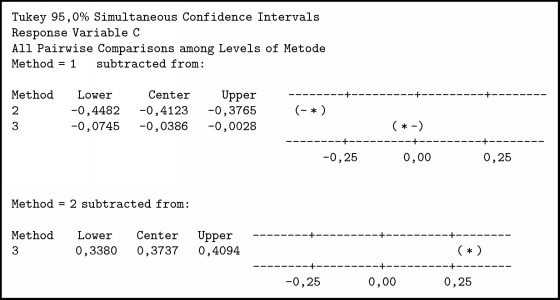
ANOVA test results on tribal population C.

**Algorithm 9:**
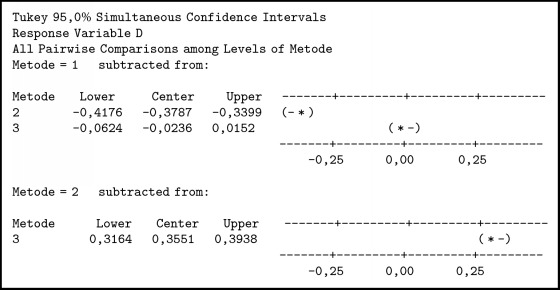
ANOVA test results on tribal population D.

**Table 1:** The results of the output on population A.

Locus: D3S1358	
Allele :1	
STR-DNA	Number of Individuals
[13]	[3]
[14]	[10]
[15]	[27]
[16]	[31]
[17]	[8]
[18]	[1]

**Table 2:** Example results of individual similarity with family reference.

**No**	**Individual1 Id**	**Relationship**	**Individual2 Id**	**Relationship**	**SCGFN**	**TFN**	**GFN**
1	0800103	mother	0800101	child	0.938856	0.525	0.798565
2	08006002	mother	0800603	child	0.822547	0.383333	0.684361
3	0800401	mother	0800403	child	0.923285	0.433333	0.747206
4	0800903	mother	0800902	child	0.931517	0.5	0.862006
5	08002F	mother	08002C	child	0.856002	0.333333	0.614684
6	08002M	father	08002C	child	0.838193	0.533333	0.738193
7	0800901	father	0800902	child	0.872816	0.566667	0.736653
8	0800102	father	0800101	child	0.850089	0.383333	0.703589
9	0800402	father	0800403	child	0.848397	0.533333	0.781071
10	08006001	father	0800603	child	0.818216	0.516667	0.739065

**Table 3:** Example results of two individuals in the same tribe.

**No**	**Individu1 Id**	**Tribe**	**Individu 2 Id**	**Tribe**	**SCGFN**	**TFN**	**GFN**
1	MT097	C	MT104	C	0.944695	0.85	0.911584
2	TRJ19	D	TRJ22	D	0.934938	0.6	0.847523
3	JT11	A	JT13	A	0.903104	0.6	0.746172
4	MW132	B	MW135	B	0.869267	0.216667	0.694363
5	JT11	A	JT12	A	0.856067	0.333333	0.669071
6	JT11	A	JT17	A	0.890797	0.233333	0.609616
7	MT021	A	MT028	A	0.924174	0.333333	0.689898
8	MT019	B	MT036	B	0.873376	0.283333	0.607031
9	MW128	B	MW123	B	0.886405	0.366667	0.671862
10	MT017	C	MT018	C	0.878135	0.333333	0.66973

**Table 4:** The result of similarity values with a certain tribal population.

**Tribe**	**SCGFN**	**TFN**	**GFN**
A	0,79	0,45	0,714639375
B	0,81	0,46	0,7615544
C	0,8071442	0,44999925	0,73734225
D	0,846345667	0,64	0,794088667

## Data Availability

The STR-DNA data used to support the findings of this study are available from the corresponding author upon request.
